# The Effect of Lipopolysaccharide-Stimulated Adipose-Derived Mesenchymal Stem Cells on NAFLD Treatment in High-Fat Diet-Fed Rats

**DOI:** 10.5812/ijpr-134807

**Published:** 2023-06-24

**Authors:** Reza Afarin, Fereshteh Aslani, Shahla Asadizade, Bahar Jaberian Asl, Mehrnoosh Mohammadi Gahrooie, Elham Shakerian, Akram Ahangarpour

**Affiliations:** 1 Cellular and Molecular Research Center, Medical Basic Sciences Research Institute, Ahvaz Jundishapur University of Medical Sciences, Ahvaz, Iran; 2 Diabetes Research Center, Health Research Institute, Ahvaz Jundishapur University of Medical Sciences, Ahvaz, Iran

**Keywords:** NAFLD/NASH, TGF-β, ADSCs, Lipopolysaccharide, Inflammation

## Abstract

**Background:**

Nonalcoholic fatty liver disease (NAFLD) and nonalcoholic steatohepatitis (NASH) are 2 common liver diseases that currently lack effective treatment options.

**Objectives:**

This study aimed to investigate the effect of lipopolysaccharide (LPS)-stimulated adipose-derived stem cells (ADSCs) on NAFLD treatment in an animal model.

**Methods:**

Male Wistar rats were fed a high-fat diet (HFD) to induce NAFLD for 7 weeks. The rats were then categorized into 3 groups: Mesenchymal stem cell (MSC), MSC + LPS, and fenofibrate (FENO) groups. Liver and body weight were measured, and the expression of genes involved in fatty acid biosynthesis, β-oxidation, and inflammatory responses was assessed.

**Results:**

Lipopolysaccharide-stimulated ADSCs were more effective in regulating liver and body weight gain and reducing liver triglyceride (TG) levels compared to the other groups. Treatment with LPS-stimulated ADSCs effectively corrected liver enzymes, including alanine aminotransferase (ALT) and aspartate aminotransferase (AST), and lipid factors, including low-density lipoprotein cholesterol (LDL-C) and high-density lipoprotein cholesterol (HDL-C) values, better than treatment with both FENO and MSCs. ADSCs + LPS treatment significantly decreased transforming growth factor β (TGF-β) and genes associated with inflammatory responses. Additionally, there was a significant reduction in reactive oxygen species (ROS) levels in the rats treated with ADSCs + LPS.

**Conclusions:**

Lipopolysaccharide-stimulated ADSCs showed potential in alleviating NAFLD by reducing inflammatory genes and ROS levels in HFD rats, demonstrating better results than treatment with ADSCs and FENO groups alone.

## 1. Background

Nonalcoholic fatty liver disease (NAFLD) is a prevalent, long-standing liver injury estimated to affect 20% to 30% of the population in Europe and the USA (1, 2). However, there is currently no effective treatment for NAFLD, increasing the risk of developing nonalcoholic steatohepatitis (NASH), cirrhosis, and liver cancer (3). The onset of NAFLD is associated with genetic and environmental factors, such as being overweight, having high blood pressure, aging, and having an elevated triglyceride level (4). Nonalcoholic fatty liver disease can arise from liver insulin resistance, lipotoxicity, impaired glucose homeostasis, accumulation of reactive oxygen species (ROS), and chronic inflammation, playing a crucial role in its progression (5, 6). As NAFLD worsens, it may progress to NASH, which is characterized by an increase in the secretion of cytokines contributing to inflammatory responses, such as IL-6, IL-1β, and tumor necrosis factor α (TNF-α) (7). Tumor necrosis factor α, a pro-inflammatory mediator, is released from adipose tissue and liver cells, such as hepatocytes and Kupffer cells, and promotes glucose uptake by repressing glucose transporter type 4 (GLUT4) expression. It also stimulates lipolysis, leading to an increase in free fatty acids (FFAs) by suppressing the expression of adipocyte genes (8, 9). Furthermore, NAFLD can worsen and develop into hepatic fibrosis by activating the Kupffer cells. Given the lack of effective treatments for NAFLD and NASH, there is a pressing need to develop new therapeutic strategies (10). The complex role of IL-6 in liver disease is associated with increased susceptibility to liver damage, stimulation of hepatocyte apoptosis, and promotion of insulin resistance, thereby contributing to the development of liver fibrosis (11). In addition, upstream mediators, such as transforming growth factor β (TGF-β), IL-6, and IL-1β (which are dependent on TNF-α), are implicated in the development of liver fibrosis (12, 13). The imbalance of these factors may lead to the development of hepatic fibrosis. Transforming growth factor β1 is the primary contributing factor in the development of hepatic fibrosis, as it activates hepatic stellate cells and promotes the production of extracellular matrix proteins (14, 15).

The role of peroxisome proliferator-activated receptors alpha and gamma (PPARα/γ) in lipid and glucose metabolism make them potential targets for the treatment of NAFLD (16). Peroxisome proliferator-activated receptors alpha is a crucial transcription factor that regulates genes involved in fatty acid β-oxidation in the peroxisome and mitochondria, thus playing a role in hyperlipidemia. One important gene affected by PPARα is carnitine palmitoyltransferase I (CPT-1α), which is involved in the pathogenesis of NAFLD. On the other hand, PPARγ promotes adipogenesis and enhances the uptake of fatty acids into adipocytes while overexpressing genes related to fatty acid mobilization (17, 18). However, because PPARγ stimulates fatty acid and glucose accumulation in cells rather than consumption, it is necessary to target the activity of these 2 factors together to treat metabolic diseases such as fatty liver (19, 20).

The sterol regulatory element binding protein-1c (SREBP-1c) is a key player in the metabolism of FFAs, induction of lipogenesis (by regulating several lipogenic genes), and inflammatory responses (21). Evidence suggests that SREBP-1c is an important factor in upregulating the expression of its downstream genes, such as fatty acid synthase (FAS) and acetyl-CoA carboxylase (ACC), contributing to the accumulation of FFAs in the liver (22). 

Fenofibrate (FENO) is a first-line medication commonly used to lower triglyceride levels. It acts as a nuclear receptor agonist of PPARα, contributing to the regulation of carbohydrate and lipid metabolism, induction of lipoprotein lipase activity, and clearance of lipoprotein remnants. However, it may also increase the excretion of cholesterol from bile, which can increase the risk of developing gallstones (23).

Mesenchymal stem cells (MSCs) are long-lived cells with self-renewal capability, and they represent an optimistic treatment strategy for NAFLD (24). Mesenchymal stem cells can be obtained from a variety of sources, including bone marrow, adipose tissue, and umbilical cord. Among them, adipose-derived stem cells (ADSCs) have gained attention for their potential to directly or indirectly repair various tissues (25). Adipose-derived stem cells can also improve NAFLD by enhancing the expression of genes involved in fatty acid oxidation while suppressing genes involved in adipogenesis, thus helping to alleviate both NAFLD and metabolic syndrome (26, 27). 

Lipopolysaccharide (LPS), a component of gram-negative bacterial cell walls, is likely to stimulate cells that contribute to inflammatory responses (such as macrophages and neutrophils) and pro-inflammatory factors (such as IL-1β, IL-6, and TNF-α) through its immune receptor (toll-like receptor 4 (TLR4)) (28). Recent studies suggest that LPS-stimulated MSCs may release anti-inflammatory cytokines during inflammation (29).

## 2. Objectives

Due to the high prevalence of NAFLD worldwide and the absence of effective treatments, finding a remedy for this disease is crucial. To address this, we conducted a study to investigate the effects of LPS-stimulated MSCs on the histological and metabolic characteristics of Wistar rats with fatty livers induced by a high-fat diet (HFD). Additionally, we examined the molecular mechanisms underlying the actions of LPS-stimulated MSCs by evaluating the expression of lipid regulatory-related genes.

## 3. Methods

### 3.1. Preparation of the High-Fat Emulsion

The adoption of a high-fat emulsion diet-induced NASH, and its composition are detailed in Table 1. The emulsion was prepared according to the description provided by Zou et al. (30). The emulsion was composed of 75% fat, 9% carbohydrates, and 14% whole milk powder as a source of protein to provide energy. The emulsion was kept at 4°C and thoroughly mixed and heated in a water bath at 42°C daily prior to use.

**Table 1.** The Macronutrient Composition and Caloric Content of a High-Fat Emulsion

High-Fat Emulsion Components	Amount
**Corn oil (g) **	400
**Saccharose (g) **	150
**Total milk powder (g) **	80
**Cholesterol (g) **	100
**Sodium deoxycholate (g) **	10
**Tween 80 (g) **	36.4
**Propylene glycol (g) **	31.1
**Vitamin mixture (g) **	2.5
**Cooking salt (g) **	10
**Mineral mixture (g) **	1.5
**Distilled water (mL) **	300
**Total energy (kcal/L) **	4342

### 3.2. Isolation and Cultivation of ADSCs

The inguinal adipose tissues of 7-week-old rats were isolated, and the blood vessels and lymph nodes were removed. The adipose tissues were then washed with phosphate-buffered saline (PBS) twice, chopped into small pieces, and transferred to a centrifuge tube (50 mL) containing 1% type I collagenase. The tubes were incubated at 37°C for 60 minutes, after which DMEM-low glucose medium supplemented with 10% FBS was added. The digested tissues were centrifuged for 15 minutes at 1500 rpm, and the resulting cells were cultured in culture flasks (25 cm) (25).

### 3.3. Induction of ADSCs with LPS

To investigate the effect of LPS on the differentiation of ADSCs, different dilutions of LPS were prepared by dissolving them in serum-free DMEM. Adipose-derived stem cells were then pretreated with LPS at a concentration of 1 μg/mL (31). After removing LPS, the cells were cultured in either serum-free or inducing medium to perform differentiation assays and evaluate their differentiation potential.

### 3.4. DiFFerentiation Assays of ADSCs

Adipose-derived stem cells from passages 3 - 5 were used for all experiments. Osteogenic and adipogenic differentiation of ADSCs was induced by culturing them in BN_0012.4 and BN_0012.5 media, respectively, following the manufacturer's instructions (Bioidea, Tehran, Iran). For osteogenic differentiation, ADSCs were seeded at a density of 20 000 cells/mL and cultured in the osteogenic medium for 3 weeks. Similarly, for adipogenesis differentiation, ADSCs were cultured in the adipogenic medium for 21 days. The cells were then observed and analyzed using a confocal microscope.

### 3.5. Ascertaining the ADSC Surface Markers

For flow cytometry analysis, ADSCs from the third passage were trypsinized with 0.025% trypsin and 0.02% EDTA, washed twice with PBS, and then stained with fluorochrome-conjugated monoclonal antibodies following the manufacturer's protocol. Specifically, FITC-conjugated mouse anti-human antibodies were used for CD34 and CD45, while PE-conjugated mouse anti-human antibodies were used for CD44 and CD105 (all antibodies were obtained from eBioscience, San Diego, CA, USA). The stained cells were analyzed using a BD FACSLyric instrument (Becton Dickinson, San Diego, CA, USA), and approximately 20 000 events were recorded for each sample. The resulting data were analyzed using FlowJo software.

### 3.6. Animals and Experimental Design

This animal-based experiment was conducted using 200- to 220-g adult male rats obtained from the Experimental Animal Center at Ahvaz Jundishapur University of Medical Sciences. The rats were placed in quarantine for 1 week to acclimatize to the environment before the commencement of experiments. During the experiment, the rats were kept in open cages in a disinfected environment with 25 ± 3°C temperature and 55% ± 8% humidity and a 12-hour light-dark cycle.

The Ethics Committee of Ahvaz Jundishapur University of Medical Sciences approved the experiments and confirmed that they were conducted in accordance with the established regulations for animal research. Initially, 40 rats were randomly selected and divided into 2 groups: The normal control group (n = 8) and the high-fat emulsion group (n = 32). Throughout the experiment, all animals followed a standard diet.

The high-fat emulsion group received a daily oral gavage of high-fat emulsion (10 mL/kg) and had access to drinking water containing 18% saccharose to induce NASH, and the normal control group received 0.5% CMC solution. After 7 weeks, 2 rats from the normal control group and 4 rats from the high-fat emulsion group were randomly selected and sacrificed to evaluate the progression of NAFLD/NASH. Once a successful high-fat-induced model was confirmed by analyzing their livers in the pathology laboratory, the pharmacological treatments were administered for 6 weeks, starting in the eighth week. The animals that were fed high-fat emulsion were then divided into 4 groups: (1) the high-fat emulsion group (n = 7) that received only high-fat emulsion; (2) the FENO group (n = 7) that received high-fat emulsion + FENO (100 mg/kg of body weight); (3) the transduced MSC group (n = 7) that received high-fat emulsion + 1.5 million transduced MSCs dissolved in 0.1- to 0.2-mL PBS injected intraperitoneally (IP); and (4) the LPS-stimulated MSC with high-fat emulsion (MSC + LPS) group (n = 7) that received high-fat emulsion + LPS-stimulated MSCs (100 mg/kg of body weight). After treatment, the rats were fasted for 24 hours and injected with a high dose of ketamine-xylazine. Blood samples were collected, and their livers were washed with normal saline and weighed to calculate the liver index (liver weight/body weight × 100). Small sections of liver tissue were frozen in liquid nitrogen (-180°C) for gene expression analysis, while larger pieces were submerged in a 10% formalin solution for histopathological investigation.

### 3.7. Biochemical Measurements

Liver enzymes (including alanine aminotransferase (ALT) and aspartate aminotransferase (AST)), high-density lipoprotein cholesterol (HDL-C), and low-density lipoprotein cholesterol (LDL-C) lipid profiles were assessed using the Roche 6000 autoanalyzer.

### 3.8. Gene Expression Investigation

The expression of genes, such as IL-6, IL-1β, TNF-α, TGF-β, and monocyte chemoattractant protein 1 (MCP-1), was examined using real-time polymerase chain reaction (PCR). Total RNA was extracted from frozen liver tissue using RNA kits (Yekta Tajhiz, Iran) following the protocol. Reverse transcription was carried out using PrimeScript RT reagent kits (Amplicon, USA). Real-time PCR was performed using an ABI Applied Biosystems' QuantStudio 3 Real-Time PCR System. The mRNA expression levels were normalized to the expression of glyceraldehyde 3-phosphate dehydrogenase (GAPDH) as an internal standard. Relative quantification was performed using Applied Biosystems software. The primer sequences were designed and then bought by SinaClon Company in Iran (Table 2).

**Table 2.** Primer Sequences Used for Gene Expression Analysis: The Amount of Each Gene Was Normalized to the Amount of Glyceraldehyde 3-Phosphate Dehydrogenase

Gene	Forward Primer	Reverse Primer
**SREBP-1c **	TCTTGACCGACATCGAGACAT	CCTGTGTCTCCTGTCTCACC
**FAS **	CCCGGACCCAGAATACCAAG	TCTTCAAGTCACACGAGGTG
**ACC**	TTAAGGGGTGAAGAGGGTGC	CACTTCCAAAGACCTAGCC
**PPARγ**	CGAGTGTGACGACAAGGTGA	ACGCTTCTTCAATCTGTCTG
**PPARα**	TGGTGCATTTGGGCGTATCT	CACGAGCGCTAAGCTGTGA
**CPT-1α**	AGCCCTGAGACAGACTCACA	ATCACGAGGGTCCGTTTTCC
**IL-1β**	TGCCACCTTTGACAGTGATG	TGATGTGCTGCTGCGAGATT
**IL-6**	CCAGTTGCCTTCTTGGGACT	TGCCATTGCACAACTCTTTC
**TNF-α**	ATGGGCTCCCTCTCATCAGT	GCTTGGTGGTTTGCTACGAC
**NOX1**	AGGCTCCAGACCTCCATTGA	AAGGCAAGGCAGTTCCGAG
**NOX2**	GGCATTCGTAGTACAGCTCA	ATTGGTCCTCGGGAGTCAGA
**NOX4**	TGGCCAACGAAGGGGTTAAA	ACACAATCCTAGGCCAACA
**TGF-β1**	CTGCTGACCCCCACTGATAC	GGGGCTGATCCCGTTGATT
**GAPDH**	CTCTCTGCTCCTCCCTGTTC	CGATACGGCCAAATCCGTTC

Abbreviations: SREBP-1c, sterol regulatory element binding protein-1c; FAS, fatty acid synthase; ACC, acetyl-CoA carboxylase; PPARγ, peroxisome proliferator-activated receptors gamma; PPARα, peroxisome proliferator-activated receptors alpha; CPT-1α, carnitine palmitoyltransferase I; TNF-α, tumor necrosis factor α; NOX1, NADPH oxidase 1; TGF-β1, transforming growth factor β1; GAPDH, glyceraldehyde 3-phosphate dehydrogenase.

### 3.9. Histopathological Examination

Liver tissue samples were dehydrated using gradient alcohol and embedded in paraffin wax. Hepatic steatosis and inflammation were assessed by staining 6- to 7-μm thick sections with hematoxylin and eosin (H&E) and Masson trichrome staining methods. A skilled liver pathologist examined the histopathological changes, and the severity of steatosis, inflammation, and fibrosis was evaluated using the NASH activity score (NAS), as described by Kleiner et al. and Liang et al. (32, 33). 

### 3.10. Statistical Analysis 

Statistical analysis was conducted using GraphPad Prism version 9 (GraphPad Software, USA). A 1-way analysis of variance (ANOVA) was used to compare the data, followed by a Tukey post hoc test. P values less than 0.05 were considered statistically significant.

## 4. Results

### 4.1. Determination of the Phenotypes of ADSCs

**Figure 1.**
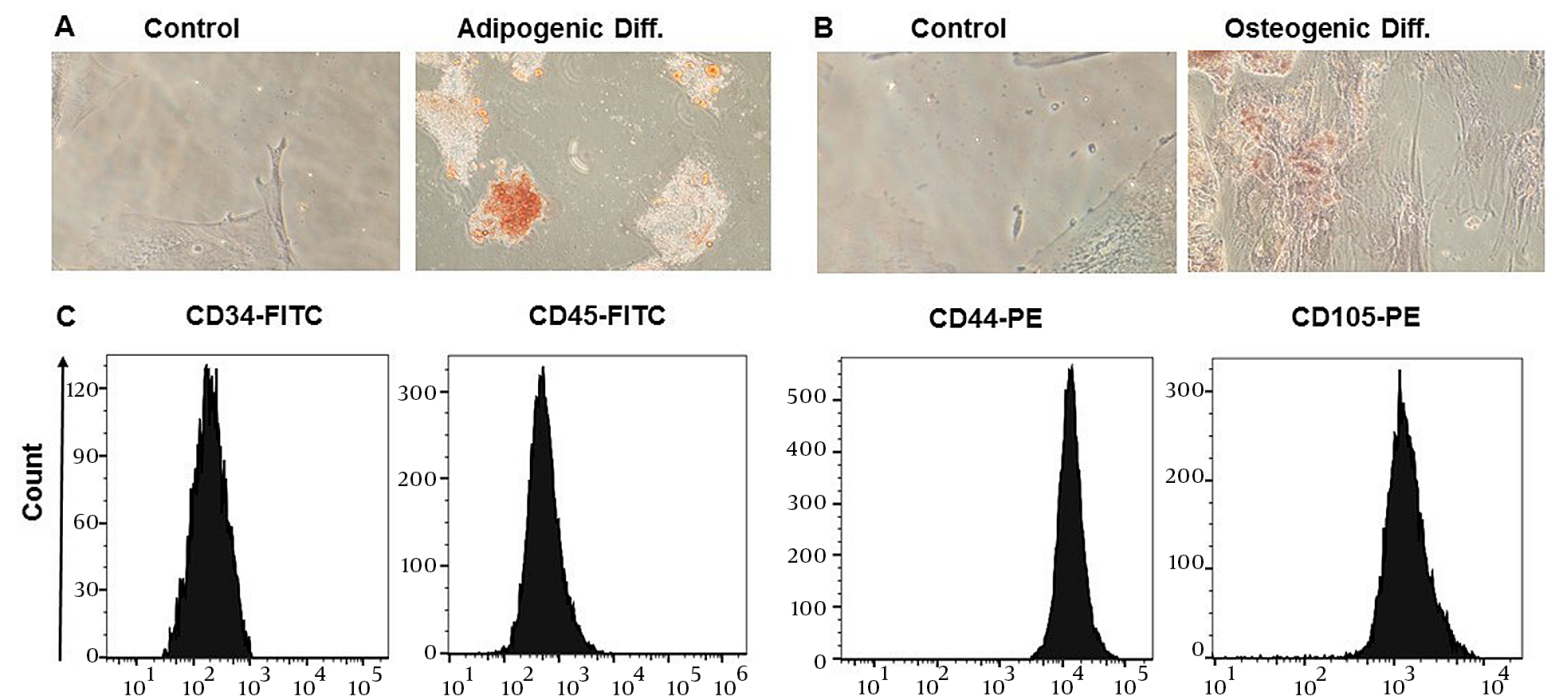
Immunophenotyping and differentiation potentials of adipose-derived stem cells (ADSCs). A, oil red O staining of ADSCs, intracellular lipid accumulation-stained bright red in adipocytes at day 21; B, alizarin red S staining of ADSCs, calcium deposition-stained bright orange-red in osteocytes at day 21; C, flow cytometry analysis of surface markers shows that ADSCs express CD44 and CD105––but CD34 and CD45 in a downregulated manner.

To ascertain the phenotypes of ADSCs, an evaluation was conducted in the laboratory to examine their surface marker expression and differentiation potential, according to the guidelines provided by the International Stem Cell Association (34). Adipose-derived stem cells were obtained from the third passage after isolation from adipose tissue and displayed fibroblast-like cells capable of adhering to flasks. Over the course of 21 days, ADSCs were cultivated in an adipogenic differentiation medium, and oil red O was used to induce adipogenic differentiation, allowing for the visualization of lipid droplet accumulation (Figure 1A). Additionally, the cells were stained with alizarin red to determine if they had differentiated into osteoblasts, which would be shown by an increase in calcium deposition (Figure 1B). Flow cytometry analysis was performed to determine the specific surface markers of ADSCs, revealing that they were positive for CD44 and CD105 (which are MSC surface markers) and negative for CD45 and CD34 (which are hematopoietic stem cells and monocyte-macrophage markers), respectively (Figure 1C).

### 4.2. Making a Comparison of Body Weight and Liver Index Following Treatment

**Figure 2.**
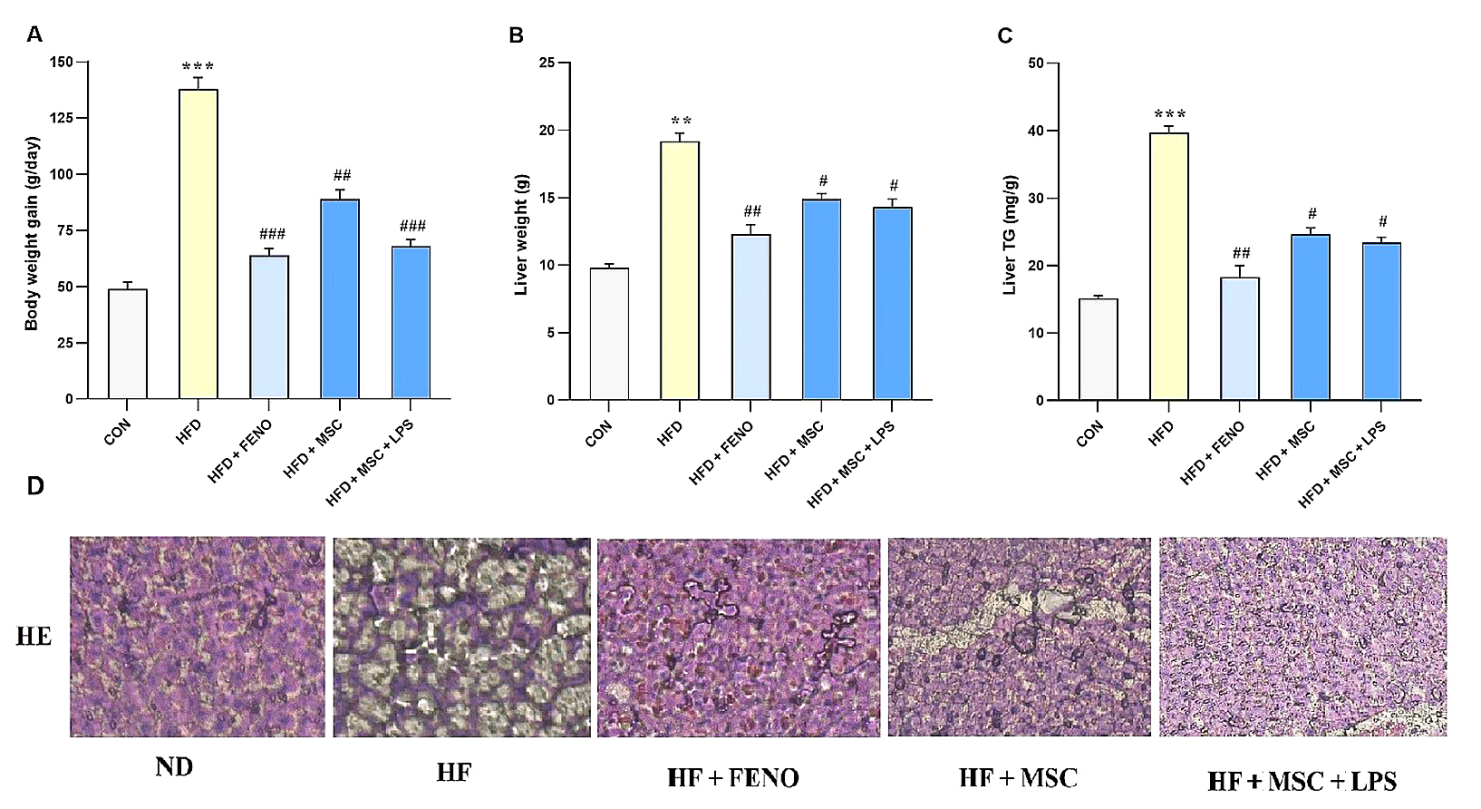
A, body weight gain; B, liver weight; and C, liver triglyceride in the high-fat-induced NASH model before and after treatment with FENO and MSCs. The mean and SD (n = 8) values are provided. Significant differences between HFD and CON are indicated by ** P < 0.01; *** P < 0.001, and significant differences between HFD and other groups are indicated by # P < 0.05; ## P < 0.01; ### P < 0.001; D, histopathological analysis of the NASH model after therapy with FENO, MSCs, and MSCs + LPS. Typical images of × 100-magnified HE-stained liver tissue from various treatment groups. Abbreviations: NASH, nonalcoholic steatohepatitis; FENO, fenofibrate; MSCs, mesenchymal stem cells; HFD, high-fat diet; CON, control; LPS, lipopolysaccharide; HE, hematoxylin and eosin; ND, normaldiet.

Regarding Figure 2, it is noted that there were no significant changes in body weights at the start of the experiment. However, rats fed with a high-fat emulsion for 12 weeks exhibited significant increase in body weight (P < 0.001; Figure 2A), liver weight (P < 0.01; Figure 2B), and liver triglyceride (P < 0.001, Figure 2C) compared to the normal control group. Over the subsequent 6 weeks, the increase in body weight was normalized by treatment with FENO as standard therapy (P < 0.001; Figure 2A), MSCs (P < 0.01; Figure 2A), and LPS-stimulated MSCs (P < 0.001; Figure 2A). Moreover, liver weight and liver triglyceride were significantly reduced by FENO, MSCs, and LPS-stimulated MSCs (P < 0.01, 0.05, 0.01 and P < 0.01, 0.05, 0.05, respectively; Figure 2B and C).

### 4.3. The Effect of FENO and ADSC Treatment on Liver Enzymes and Lipid Profiles in the NAFLD Model

**Figure 3.**
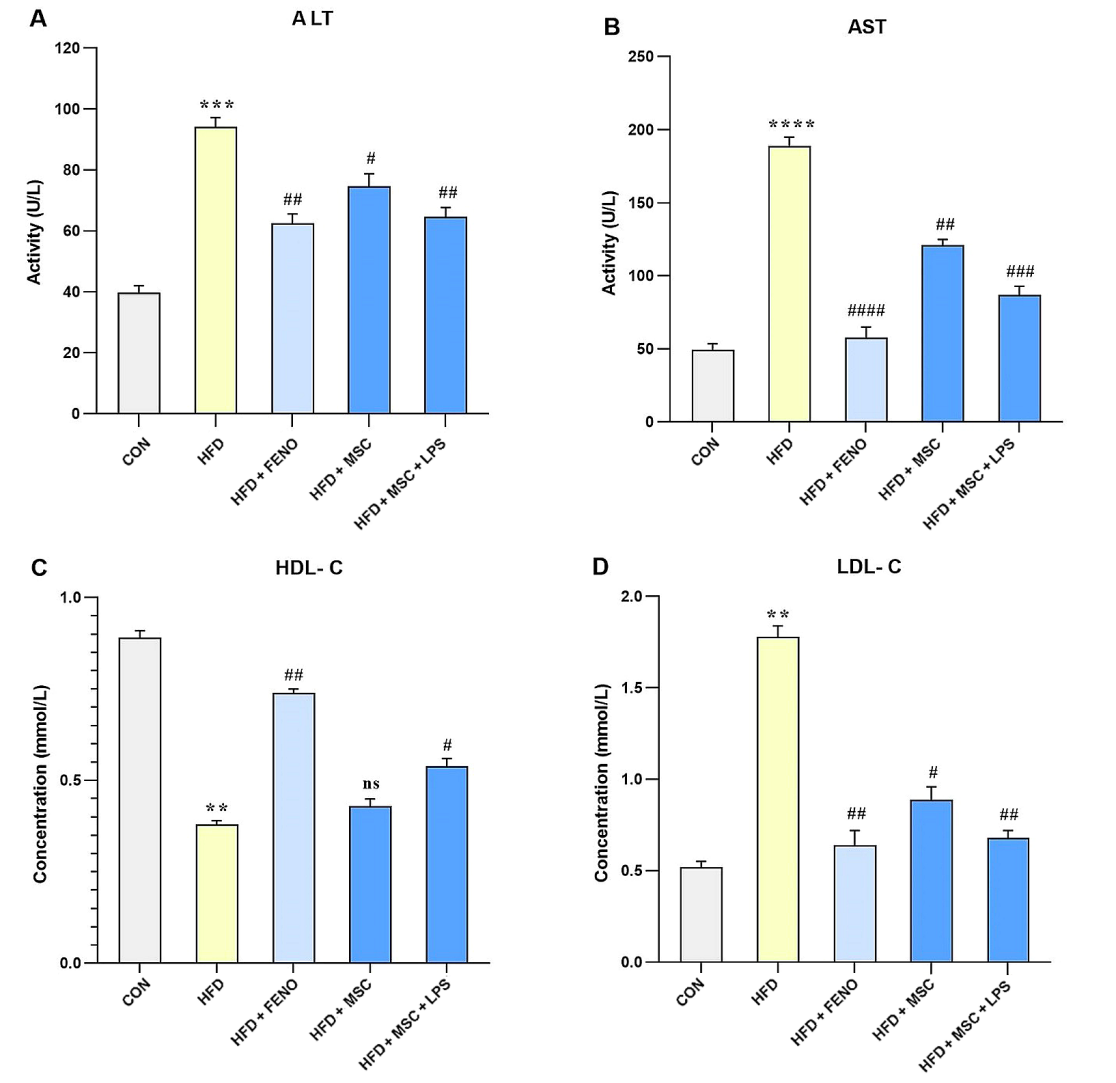
The values represent the mean and SD of 7 rats. An ANOVA and Tukey-Kramer multiple comparison tests were used to examine differences between the groups. Significant differences between HFD and CON are indicated by ** P < 0.01; *** P < 0.001; **** P < 0.0001, and significant differences between HFD and other groups are indicated by # P < 0.05; ## P < 0.01; ### P < 0.001; #### P < 0.0001. Abbreviations: ANOVA, analysis of variance; ALT, alanine aminotransferase; AST, aspartate aminotransferase; HDL-C, high-density lipoprotein cholesterol; LDL-C, low-density lipoprotein cholesterol; HFD, high-fat diet; CON, control; FENO, fenofibrate; MSC, mesenchymal stem cell; LPS; lipopolysaccharide.

In this study, the biomarkers of liver injury (such as ALT and AST) and lipid factors (including HDL-C and LDL-C) were evaluated to assess the effects of various treatments on NAFLD. The results showed that feeding rats a high-fat emulsion led to significant increases in ALT (P < 0.001; Figure 3A), AST (P < 0.0001; Figure 3B), and LDL-C (P < 0.01; Figure 3D), while HDL-C levels decreased (P < 0.01; Figure 3C) compared to the normal control group. However, treatment with FENO led to a decline in ALT (P < 0.01; Figure 3A), AST (P < 0.001; Figure 3B), and LDL-C (P < 0.01; Figure 3D), as well as an increase in HDL-C levels (P < 0.01; Figure 3C) compared to the high-fat emulsion group. Similarly, treatment with MSCs resulted in a reduction in serum levels of ALT (P < 0.05; Figure 3A), AST (P < 0.01; Figure 3B), and LDL-C (P < 0.05; Figure 3D) but did not significantly modify the elevated level of HDL-C. Furthermore, stimulation with LPS enhanced the effects of MSC treatment on the reduction of ALT (P < 0.01; Figure 3A), AST (P < 0.001; Figure 3B), and LDL-C (P < 0.01; Figure 3D), as well as an increase in HDL-C levels (P < 0.05; Figure 3C).

### 4.4. Regulation of Lipid-Related Gene Expression Following FENO and ADSC Treatment 

**Figure 4.**
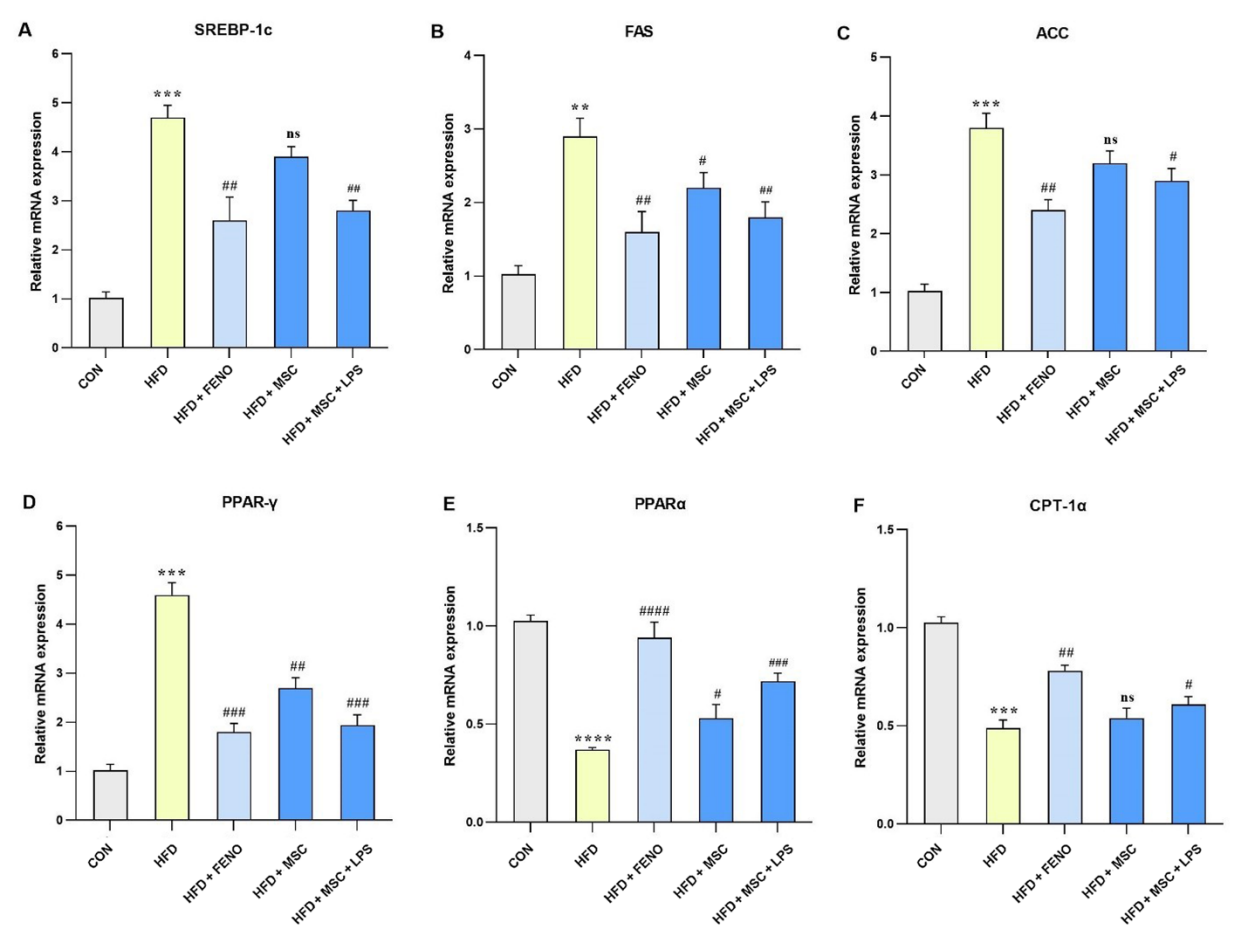
Gene expression levels pertaining to lipid-related genes. Using quantitative real-time PCR, hepatic mRNA levels were measured and normalized to GAPDH mRNA expression. The values are shown as the mean and SD of fold changes compared to the CON. An ANOVA and Tukey-Kramer tests were used for multiple comparisons to evaluate between-group differences. Significant differences between HFD and NC are indicated by **P < 0.01; *** P < 0.001; **** P < 0.0001, and significant differences between HFD and other groups are indicated by # P < 0.05; ## P < 0.01; ### P < 0.001; #### P < 0.0001. Abbreviations: SREBP-1c, sterol regulatory element binding protein-1c; FAS, fatty acid synthase; ACC, acetyl-CoA carboxylase; PPARγ, peroxisome proliferator-activated receptors gamma; PPARα, peroxisome proliferator-activated receptors alpha; CPT-1α, carnitine palmitoyltransferase I; CON, control; HFD, high-fat diet; FENO, fenofibrate; MSC, mesenchymal stem cell; LPS, lipopolysaccharide; PCR, polymerase chain reaction; GAPDH, glyceraldehyde 3-phosphate dehydrogenase; ANOVA, analysis of variance.

The study found a significant increase in the expression levels of hepatic mRNA genes involved in fatty acid biosynthesis, namely SREBP-1c (P < 0.001; Figure 4A), FAS (P < 0.01; Figure 4B), and ACC (P < 0.001; Figure 4C), as well as genes with important roles in fatty acid β-oxidation, such as PPARγ and CPT-1α (P < 0.001; Figure 4D and F) and PPARα (P < 0.0001; Figure 4E), following an HFD in the high-fat emulsion group. The FENO treatment resulted in a reduction in the levels of SREBP-1c, FAS, and ACC (P < 0.01; Figure 4A, B, and C) and PPARγ (P < 0.001; Figure 4D) while inducing an upward trend in the expression levels of PPARα (P < 0.001; Figure 4E) and CPT-1α (P < 0.01; Figure 4F). The MSC treatment had varying effects on the expression levels of these genes. Specifically, the expression levels of SREBP-1c and ACC were not significantly reduced compared to FAS and PPARγ that decreased (P < 0.05 and P < 0.01, respectively; Figure 4B and D). On the other hand, the LPS-stimulated MSC treatment led to significant downturns in SREBP-1c and FAS (P < 0.01; Figure 4A and B), as well as ACC (P < 0.05; Figure 4C) and PPARγ (P < 0.001; Figure 4D). Conversely, the expression levels of PPARα (P < 0.001; Figure 4E) and CPT-1α (P < 0.05; Figure 4F) increased following the LPS-stimulated MSC treatment.

### 4.5. Reduction of Oxidative Stress and Related Gene Expression after the FENO and ADSC Treatment

**Figure 5.**
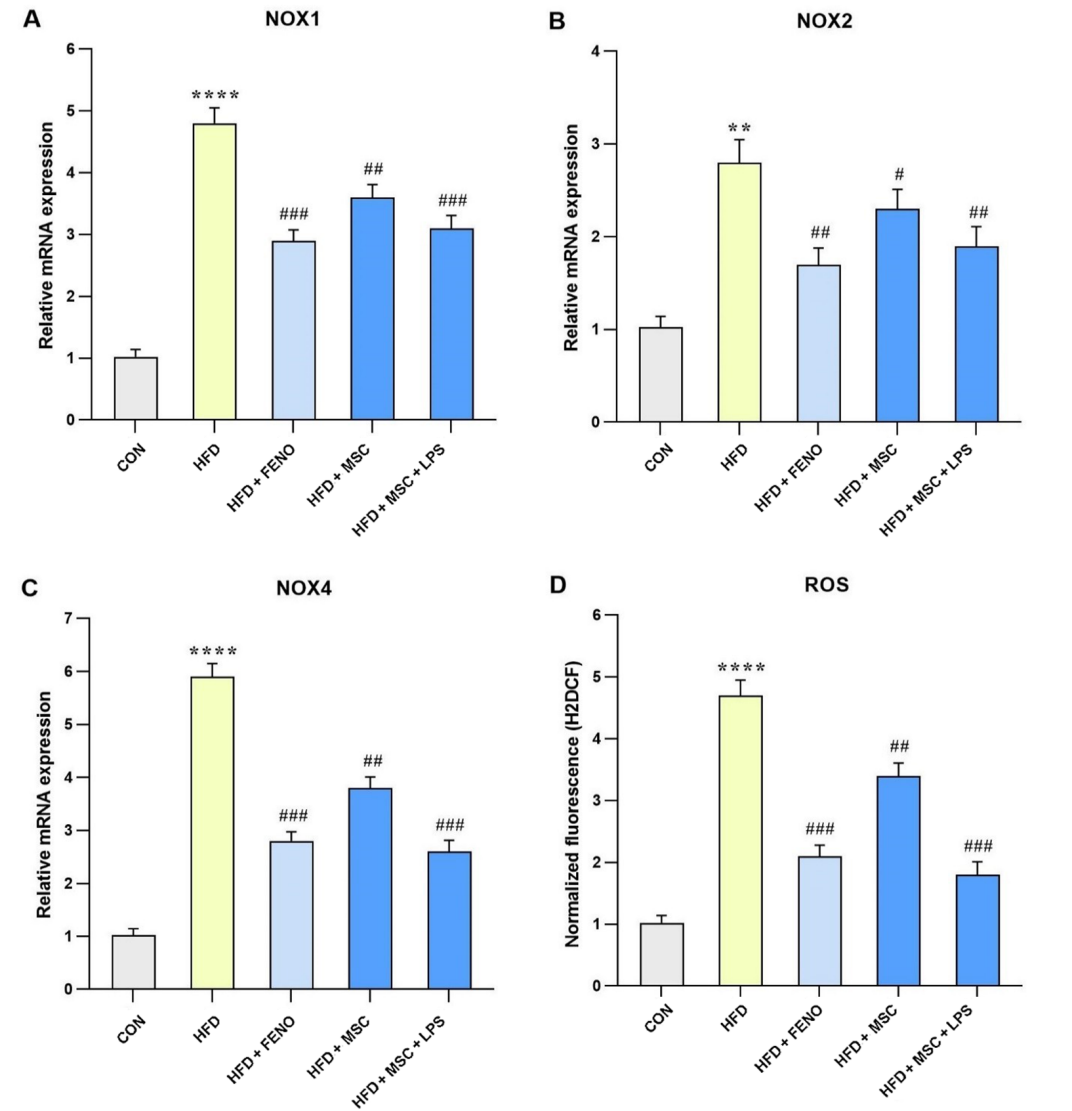
Gene expression levels of oxidative stress in response to MSC group (MSC, MSC + LPS) treatment. Using quantitative real-time PCR, hepatic mRNA levels were evaluated and normalized to GAPDH mRNA expression. The values are given as the mean and SD of fold changes compared to the normal control group. An ANOVA and Tukey-Kramer multiple comparisons tests were used to examine between-group differences. Significant differences between HFD and NC are indicated by ** P < 0.01; **** P < 0.0001, and significant differences between HFD and other groups are indicated by # P < 0.05; ## P < 0.01; ### P < 0.001. Abbreviations: NOX1, NADPH oxidase 1; ROS, reactive oxygen species; CON, control; HFD, high-fat diet; FENO, fenofibrate; MSC, mesenchymal stem cell; LPS, lipopolysaccharide; MSC, mesenchymal stem cell; LPS, lipopolysaccharide; PCR, polymerase chain reaction; GAPDH, glyceraldehyde 3-phosphate dehydrogenase; ANOVA, analysis of variance.

The data presented in Figure 4 compares gene expression levels involved in oxidative stress between the HFD and the control groups, as well as between the FENO and MSC groups, compared to the high-fat emulsion group. In the high-fat emulsion group, there was a substantial increase in the expression levels of NADPH oxidase 1 (NOX1), NOX4, and ROS (P < 0.0001; Figure 5A, C, and D) and NOX2 (P < 0.01; Figure 5B). In contrast, the FENO treatment led to a significant reduction in NOX1, NOX4, and ROS levels (P < 0.001; Figure 5A, C, and D) and NOX2 (P < 0.01; Figure 5B). Similarly, the MSC treatment resulted in a decline in the expression levels of NOX1, NOX4, and ROS (P < 0.01; Figure 5A, C, and D) and NOX2 (P < 0.05; Figure 5B). Notably, stimulation of MSC with LPS caused a considerable decrease in the levels of NOX1, NOX4, and ROS (P < 0.001; Figure 5A, C, and D) and NOX2 (P < 0.01; Figure 5B).

### 4.6. Reduction of Inflammatory mRNA Expression Following the FENO and ADSC Treatment

**Figure 6.**
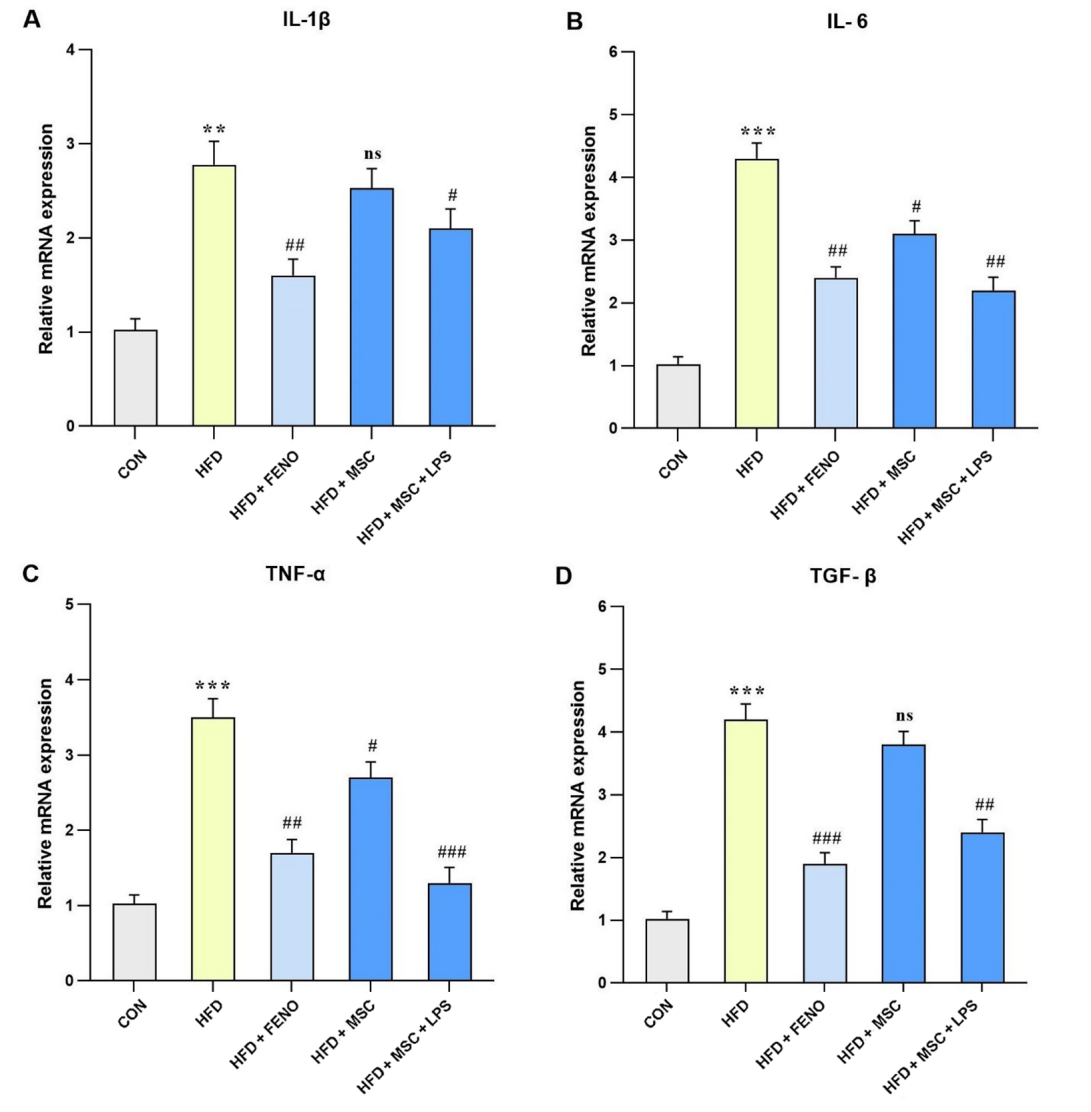
Gene expression levels of pro-inflammatory cytokines and TNF-α in response to MSC group (MSC, MSC + LPS) treatment. Using quantitative real-time PCR, hepatic mRNA levels were evaluated and normalized to GAPDH mRNA expression. The values are given as the mean and SD of fold changes compared to the CON. An ANOVA and Tukey-Kramer multiple comparisons tests were used to examine between-group differences Significant differences between HFD and NC are indicated by ** P < 0.01; *** P < 0.001, and significant differences between HFD and other groups are indicated by; ## P < 0.01; ### P < 0.001. Abbreviations: CON, control; HFD, high-fat diet; FENO, fenofibrate; MSC, mesenchymal stem cell; LPS, lipopolysaccharide; TNF-α, tumor necrosis factor α; MSC, mesenchymal stem cell; LPS, lipopolysaccharide; PCR, polymerase chain reaction; GAPDH, glyceraldehyde 3-phosphate dehydrogenase; ANOVA, analysis of variance.

Regarding the analysis of hepatic mRNA expression of pro-inflammatory genes, including IL-6, TNFα, and TGF-β (P < 0.001; Figure 6B, C, and D) and IL-1β (P < 0.01; Figure 6A), a significant increase was observed in the high-fat emulsion group. The FENO treatment resulted in a substantial reduction in the mRNA expression level of IL-1β, IL-6, and TNF-α (P < 0.01; Figure 6A, B, and C) and TGF-β (P < 0.001; Figure 6D). Moreover, in the high-fat emulsion group treated with MSC, the mRNA expression levels of IL-6 and TNF-α (P < 0.05; Figure 6B and C) decreased compared to IL-1β and TGF-β, but no significant reduction was observed in their expression levels. Notably, significant decreases were observed following MSC stimulation with LPS, whereby the expression levels of IL-6 and TGF-β (P < 0.01; Figure 6B and D), IL-1β (P < 0.05; Figure 6A), and TNF-α (P < 0.001; Figure 6C) noticeably decreased.

## 5. Discussion

Nonalcoholic fatty liver disease is a complex disorder with a multi-hit pathophysiology and is considered one of the systemic diseases where the deposition of fat in the liver is the initial contributing factor (4). The increasing prevalence of NAFLD can be attributed to lifestyle changes, such as unhealthy eating habits and high rates of obesity, regardless of excessive alcohol consumption (3). Consequently, lifestyle modification is currently the primary therapy for patients with NAFLD. Additionally, other factors contributing to this disease include oxidative stress and inflammatory responses, playing critical roles in the progression of NAFLD to NASH and liver fibrosis. If left untreated, NAFLD can lead to liver cancer and liver failure (35).

Mesenchymal stem cells have emerged as a promising therapy due to their ability to regenerate and repair damaged tissue. Furthermore, the stimulation of MSCs with LPS has been reported to increase the production of anti-inflammatory cytokines during inflammatory states. In light of these findings, we stimulated MSCs with LPS and compared them to unstimulated MSCs. Recent studies suggest that MSCs have the potential to downregulate inflammatory genes, such as IL-1β, TNF-α, and IL-6, as well as to reduce the levels of ROS (31, 36).

In the current investigation, a NAFLD rat model induced by HFD was employed to assess the efficacy of LPS-stimulated MSCs compared to FENO, a widely used hypolipidemic drug for dyslipidemia treatment. Fenofibrate, a PPARα agonist, is one of the drug targets for NAFLD due to its contribution to mitigating inflammation, glucose, and lipid homeostasis. This drug promotes hepatic lipid oxidation, resulting in a reduction in hepatic triglyceride levels (37). Fenofibrate is known to improve lipoprotein remnant clearance by reducing LDL synthesis and mildly elevating HDL levels (38). In the current investigation, rats fed an HFD exhibited elevated liver triglyceride levels and liver weight due to hepatic lipid accumulation. Our findings suggest that LPS-stimulated MSCs may enhance the activity of PPARs, a group of transcription factors that regulate lipid metabolism. The AMP-activated protein kinase (AMPK)-PPAR pathway is a crucial player in lipid metabolism. According to our results, FENO activates this pathway by inhibiting the activity of SREBP-1c, FAS, ACC, and PPARγ while elevating the expression levels of PPARα and CPT1.

Acetyl-CoA carboxylase is an important enzyme involved in both fatty acid synthesis and oxidation in hepatic cells. As a downstream target of AMPK, ACC is regulated by phosphorylation, and its expression level is reduced upon AMPK activation. This leads to a decrease in fatty acid biosynthesis while increasing the expression levels of PPARα and CPT-1, ultimately promoting fatty acid oxidation. On the other hand, SREBP-1c, FAS, and ACC are involved in de novo lipogenesis and fatty acid biosynthesis (39, 40).

According to the findings reported by Prasad et al, the suppression of inflammatory responses was found to alleviate fibrosis development in an HFD-induced NAFLD model (41). Lipid accumulation is known to initiate an inflammatory response and contribute to the progression of NAFLD to NASH and liver fibrosis. This process is thought to be associated with an increase in ROS levels, which can be mediated through the expression of NOX enzymes, including NOX1, NOX2, and NOX4. Furthermore, the induction of an inflammatory state can activate MSCs and recruit macrophages to the site of inflammation, which may lead to an exacerbated immune response via the release of MCP-1.

Inflammatory gene expression, including IL-1β, IL-6, TNF-α, and TGF-β, is intricately involved in the progression of NAFLD (42). Tumor necrosis factor α expression, for example, is thought to contribute to the progression of NAFLD to NASH by inducing molecules involved in lipid and glucose metabolism, inflammatory factors, and fibrosis in hepatocytes. Additionally, TNF-α stimulates TGF-β expression, a critical factor that plays a significant role in the processes of inflammation and fibrogenesis (43). Recent studies have shown that PPARα agonists, such as FENO, have the potential to decrease TNF-α and TGF-β expression in animal-based experiments, thus ameliorating the damage caused by NASH. However, due to the numerous chemical side effects of FENO, it is preferable to pursue new approaches to treat dyslipidemia (44).

In the in vivo study conducted by Xu et al, the synergistic effects of liraglutide and human umbilical cord MSCs on the inflammatory pathway in rats fed a high-fat and high-sucrose diet were investigated. The findings revealed that the combination therapy significantly reduced ALT and AST levels, improved liver histopathology, and alleviated liver inflammation by downregulating TLR4, nuclear factor kappa B (NF-κB), IL-6, and TNF-α while enhancing antioxidant activity. Notably, both MSCs and LPS-stimulated MSCs attenuated the production of pro-inflammatory cytokines, such as IL-6 and TNF-α, leading to a reduction in liver inflammation. Furthermore, the levels of liver enzymes, including ALT and AST, were significantly reduced (45). In a recent animal-based study conducted by Li et al. in 2021, an HFD-induced model of NAFLD was used to investigate the effects of MSCs on the biosynthesis, β-oxidation, and inflammatory pathways. This was achieved by examining the expression levels of several key genes, including IL-1β, IL-18, PPARα, CPT-1, ACC, and SREBP. The findings of this investigation demonstrated that MSC treatment effectively modulated the expression of these genes, thereby influencing the aforementioned pathways (46). The present study similarly reveals comparable results, with statistically significant outcomes observed in certain instances.

In 2021, Shen and colleagues investigated the expression of IL-1β, TNF-α, IL-6, IL-10, heme oxygenase-1(HO-1), nuclear factor erythroid 2-related factor 2 (Nrf2), and ROS levels in rats fed an HFD. They demonstrated that MSCs could regulate the changes in inflammatory gene expression and focused on the antioxidant response by examining Nrf2/H0-1 (36). However, our study shows a greater decrease in both ROS and inflammatory gene expression, which may be attributed to the regulation of NF-κB, as it exerts more significant effects.

### 5.1. Conclusions

Mesenchymal stem cells stimulated with LPS exhibit a significant reduction in the expression of inflammatory genes, including IL-1β and IL-6, and ROS levels, potentially mediated by the NF-κB pathway. Moreover, the induction of MSCs with LPS results in more robust effects. Additionally, the histopathological assessment indicated that the MSC treatment could ameliorate NAFLD-induced damages, comparable to the effects of FENO, a widely used PPARα agonist drug.
